# A *Medicago truncatula* HD-ZIP gene *MtHB2* is involved in modulation of root development by regulating auxin response

**DOI:** 10.3389/fpls.2024.1466431

**Published:** 2024-09-19

**Authors:** Wei Yan, Runze Wang, Yutong Zhang, Xiuxiu Zhang, Qin Wang

**Affiliations:** ^1^ Institute of Biotechnology, Inner Mongolia Academy of Science and Technology, Hohhot, China; ^2^ Department of Biochemistry and Molecular Biology, Binzhou Medical University, Yantai, China; ^3^ College of Grassland Resources and Environment, Inner Mongolia Agricultural University, Hohhot, China; ^4^ State Key Laboratory of Vegetation and Environmental Change, Institute of Botany, Chinese Academy of Sciences, Beijing, China

**Keywords:** auxin, HD-ZIP II, MtHB2, *Medicago truncatula*, root development

## Abstract

HD-Zip proteins are plant-specific transcription factors known for their diverse functions in regulating plant growth, development, and responses to environmental stresses. Among the *Medicago truncatula* HD-Zip II genes, *MtHB2* has been previously linked to abiotic stress responses. In this study, we conducted a functional characterization of *MtHB2* in the regulation of root growth and development. Upon auxin stimulation, expression of *MtHB2* was promptly up-regulated. Overexpression of *MtHB2* in *Arabidopsis thaliana* led to reduced primary root growth and inhibited lateral root formation. Interestingly, the transgenic plants expressing *MtHB2* exhibited differential responses to three types of auxins (IAA, NAA, and 2,4-D) in terms of root growth and development compared to the wild-type plants. Specifically, primary root growth was less affected, and lateral root formation was enhanced in the transgenic plants when exposed to auxins. This differential response suggests a potential role for *MtHB2* in modulating auxin transport and accumulation, as evidenced by the reduced sensitivity of the transgenic plants to the auxin transport inhibitor NPA and lower expression levels of auxin-related reporters such as *PIN*-*FORMED* (*PIN1*)::PIN1-GFP, *PIN3*::PIN3-GFP, *PIN7*::PIN7-GFP, and *DR5*::GFP compared to wild-type plants. Additionally, microarray analysis of the root tissues revealed down-regulation of several auxin-responsive genes in transgenic seedlings compared to wild-type plants. These findings collectively indicate that *MtHB2* plays a critical regulatory role in root growth and development by modulating auxin accumulation and response in the roots.

## 1 Introduction

The homeodomain-leucine zipper (HD-Zip) family of transcription factors unique to plants is characterized by the presence of a homeodomain (HD) and an adjacent leucine zipper (Zip) motif (Li et al., 2022a). These factors are categorized into four subfamilies (HD-Zip I, II, III, and IV) based on their sequence conservation, structural characteristics, and functions (Harris et al., 2011; Li et al., 2022a). The HD domain is responsible for specific binding to target DNA through helix III, while the leucine zipper domain’s dimerization ability is crucial for DNA binding. HD-Zip I and II proteins recognize similar pseudopalindromic binding sites (CAATNATTG), while HD-Zip III and IV proteins interact with slightly different sequences (GTAAT[G/C]ATTAC and TAAATG[C/T]A, respectively) (Ariel et al., 2007).

Different subfamilies of HD-Zip have significant functional differences. HD-Zip III proteins act as developmental regulators in various plant structures, such as the embryo, shoot, root, leaves, seeds and vasculature, while HD-Zip IV proteins are involved in determining cell fates in the epidermal layer (Brandt et al., 2014; Gu et al., 2024). HD-Zip I transcription factors are associated with abiotic and biotic stress responses, leaf and flower senescence, floral organogenesis, ripening, and responses to light conditions (Whipple et al., 2011; Zhao et al., 2014; Gong et al., 2019). HD-Zip II proteins are also involved in responses to stresses. For instance, expression studies have shown that transcripts of some members of HD-Zip II genes are up-regulated by stress (Harris et al., 2011; Li et al., 2019). Several studies revealed that HD-Zip II genes are associated with leaf chlorophyll, leaf senescence, induction of flowering, and phytohormone-mediated responses to biotic stresses (Oh et al., 2013). *HAT22*, an *Arabidopsis* HD-Zip II β gene, is up-regulated by cytokinin, and overexpression of *HAT22* reduces chlorophyll contents and accelerates leaf senescence (Köllmer et al., 2011). *ATHB2*-induced hypocotyl elongation and small leaves has been shown to be dependent on the auxin transport and response (Steindler et al., 1999; He et al., 2020). It has recently emerged that HD-Zip II proteins also regulate several aspects of plant development, including embryo patterning, meristem function, leaf polarity and carpel development (Carabelli et al., 2013; Preciado et al., 2022). However, there have been few reports on the involvement of HD-Zip proteins in the regulation of root system architecture.

Root system architecture is a crucial trait associated with the acquisition of water and nutrients in plants. Plants have developed various mechanisms to adjust their root system architecture in order to respond and adapt to constantly changing environments. Plant hormonal and environmental signals work together to regulate root system architecture (Fukaki and Tasaka, 2009). It is widely acknowledged that auxin plays a vital role in root growth and development. Auxin stands out among plant hormones due to its active and directional transport from the site of synthesis in young apical parts to distant tissues (Li et al., 2023). The PIN-FORMED (PIN) auxin efflux regulators, which control polar auxin transport, are essential for the distribution of auxin throughout the plant (Blilou et al., 2005). PIN1 is crucial for basipetal auxin transport in shoots and acropetal transport in roots. And, PIN3 and PIN7 are involved in acropetal auxin transport in roots (Blilou et al., 2005). The PIN network governs growth and patterning in *Arabidopsis* roots (Lee et al., 2020). Mutants in the *PIN* genes display impaired auxin transport, leading to reduced lateral root initiation and leaf organogenesis (Benková et al., 2003). Polar transport of auxin affects the formation of lateral roots (Casimiro et al., 2003). Previous studies have demonstrated that auxin derived from the shoot is necessary for the development of lateral roots in young seedlings (Lee et al., 2020).

Studies with transgenic plants have indicated that overexpression of HD-Zip II genes *ATHB2*, *HAT2*, *HAT1*, and *ATHB4* leads to reduced primary root growth and lateral root formation, affecting root responsiveness to exogenous auxin (Sorin et al., 2009; He et al., 2020). The precise mechanism by which HD-Zip II genes modulate auxin response and transport to regulate root growth and development warrants further investigation. Additionally, the *Medicago truncatula* HD-Zip I transcription factor HB1 (MtHB1) has been identified as regulating root architecture by inhibiting the LATERAL ORGAN BOUNDARIES DOMAIN 1 (LBD1) transcription factor within the auxin pathway (Ariel et al., 2010).

Among 14 HD-ZIP protein of *Medicago truncatula* which is the model legume plant, it was found that the MtHB2 negatively regulates abiotic stresses when expressed in *Arabidopsis* (Song et al., 2012; Li et al., 2022b; Zhang et al., 2022). It has been identified that *MtHB2* is closely related to *Arabidopsis* HD-Zip II β genes (*HAT22* and *HAT9*) (Song et al., 2012). Our research demonstrates that MtHB2 is involved in the regulation of root development, evident from the abnormalities in root growth and development observed in transgenic *Arabidopsis* plants expressing *MtHB2*. Furthermore, we highlight the role of MtHB2 in modulating root growth and development through its regulation of auxin transport and response.

## 2 Materials and methods

### 2.1 Plant material and growth conditions

*Arabidopsis thaliana* ecotype Columbia-0 (Col-0), pro*DR5::GFP* (Friml et al., 2003), pro*PIN1::PIN1-GFP* (Benková et al., 2003), pro*PIN3::PIN3-GFP* and pro*PIN7::PIN7-GFP* (Blilou et al., 2005) were obtained from the *Arabidopsis* Biological Resource Centre, Columbus, OH, USA. The vector of pSN1301:*MtHB2* which was driven by CaMV 35S was constructed and generation of transgenic plants was previously described (Clough and Bent, 1998). Homozygous transgenic lines of all plants above were selected for this study. Plants with various GFP fusions were obtained by crossing pro*DR5::GFP*, pro*PIN1::PIN1-GFP*, pro*PIN3::PIN3-GFP* and pro*PIN7::PIN7-GFP* lines with *MtHB2*-expressing transgenic plants L4.

For all *Arabidopsis* experiments, seeds underwent surface sterilization by a 1-minute incubation in 75% ethanol, followed by rinsing with sterile water, treatment with 10% (v/v) sodium hypochlorite for 15 minutes, and thorough washing with sterile water. These sterilized seeds were then sown on half-strength Murashige and Skoog (1/2 MS) agar plates supplemented with 0.8% sucrose and 0.6% agar (w/v) at pH 5.8. After stratification for 2 days at 4°C, the seeds were grown in a growth chamber under a 14-hour photoperiod at 23/20°C and a photosynthetic photon flux density of 100–120 µmol m^-2^ s^-1^.

Seeds of *Medicago truncatula* ‘Jemalong A17’ were treated by soaking in concentrated sulfuric acid for approximately 6 minutes, followed by thorough rinsing with tap water, and then sown on Petri plates containing 0.8% agar. After chilling at 4°C for a day, the seeds were germinated in darkness at 25°C for 2 days. Subsequently, seedlings with approximately 2 cm radicles were transferred to plastic buckets filled with fully aerated nutrient solution and grown in a growth chamber under a 14-hour photoperiod at 25/20°C with a photosynthetic photon flux density of 200–230 µmol m^-2^ s^-1^. To assess *MtHB2* expression in response to auxin, two-week-old seedlings were exposed to a solution containing 10 µM indole 3-acetic acid (IAA) for varying periods (0.5, 1, 2, 4, 8 hours), followed by root sampling for total RNA extraction.

### 2.2 Root growth assays

To characterize root phenotypes, 6-day-old seedlings were transferred to 1/2 MS medium with 1% (w/v) agar and grown vertically for an additional 4 days. For the analysis of root responses to auxin, 6-day-old seedlings were transferred to 1/2 MS medium with 1% (w/v) agar containing 0.1 µM 1-naphthyl acetic acid (NAA), 1 µM IAA, and 0.05 µM 2,4-dichloro-phenoxyacetic acid (2,4-D) and grown for 4 days. To study the inhibition of lateral root formation by naphthylphthalamic acid (NPA) and 2,3,5-triiodobenzoic acid (TIBA), 6-day-old seedlings were transferred to 1/2 MS medium with 1% (w/v) agar containing 0.5 µM NPA and 0.1 µM TIBA and grown for 6 days. Primary root length was measured using a ruler, and the number of lateral roots exceeding approximately ≥0.5 mm in length was documented. Each treatment involved at least 15 independent seedlings, and all experiments were conducted at least three times.

To quantify the number of lateral root primordia, the entire root was stained with methylene blue (Johnson et al., 1996), with slight modifications. The roots were fixed in a solution of absolute ethanol and glacial acetic acid (3:1, v/v) at 4°C for a minimum of 24 hours, rinsed with distilled water, and then stained with methylene blue (0.01% in distilled water) to visualize the lateral root primordia under a microscope (SZX12, OLYMPUS, Japan). Using this method, dome-shaped lateral root primordia can be observed at the later stages of development before emerging from the parental roots (Peret et al., 2009; Ni et al., 2014).

### 2.3 Confocal microscopy

Roots of 2-day-old and 6-day-old seedlings carrying pro*DR5*::*GFP*, pro*PIN1::PIN1-GFP*, pro*PIN3::PIN3-GFP*, and pro*PIN7::PIN7-GFP* were imaged using a laser confocal scanning microscope Zeiss LSM510. The fluorescence intensity was quantified using the ImageJ program on confocal sections acquired with consistent settings on the same day for all samples within an experiment.

### 2.4 Microarray analysis

Total RNA was extracted from the roots of 18-day-old wild-type and *MtHB2*-expressing transgenic plants using TRIzol reagent. The microarrays were manufactured as previously described (Yang et al., 2012), and all labeling, hybridization, and washing processes were conducted through the Affymetrix custom service (Capitalbio) following the provided protocols. Normalization was carried out as per standard Affymetrix protocols to enable sample comparison, with genes showing more than a 2-fold change considered significantly different. The study employed two biological replicates.

### 2.5 RNA extraction and real-time quantitative PCR

Total RNA was extracted using RNAiso Plus reagent (TaKaRa) and reverse-transcribed into first-strand cDNA with the PrimeScript^®^ RT reagent Kit (with gDNA eraser) (TaKaRa). Real-time quantitative PCR (RT-qPCR) was conducted using an ABI StepOne Plus instrument. The gene-specific primers utilized for RT-qPCR are detailed in **Supplementary Table S1**. The *MtActin* gene (accession No. BT141409) and *AtActin11* (accession No. NM_112046) were employed as internal controls to standardize the expression levels. Each reaction mixture consisted of 5 µL of 2×UltarSYBR Mixture (with ROX) reagent (Cwbio), 2 µL of cDNA samples, and 0.6 µL of 10mM gene-specific primers in a final volume of 10 µL. The thermal cycling program included an initial denaturation at 95°C for 10 min, followed by 40 cycles of 95°C for 30 s, 55°C for 30 s, and 72°C for 30 s. The relative expression level was determined using the comparative CT method as described by Livak and Schmittgen (2001).

### 2.6 Statistical analysis

All data were statistically analyzed using analysis of variance with the One-way ANOVA or Independent-Samples t-test through the SPSS 17.0 statistics program. Statistical significance was denoted when P < 0.05.

## 3 Results

### 3.1 Expression of *MtHB2* in *Arabidopsis* disrupts root growth and development

**Figure 1:**
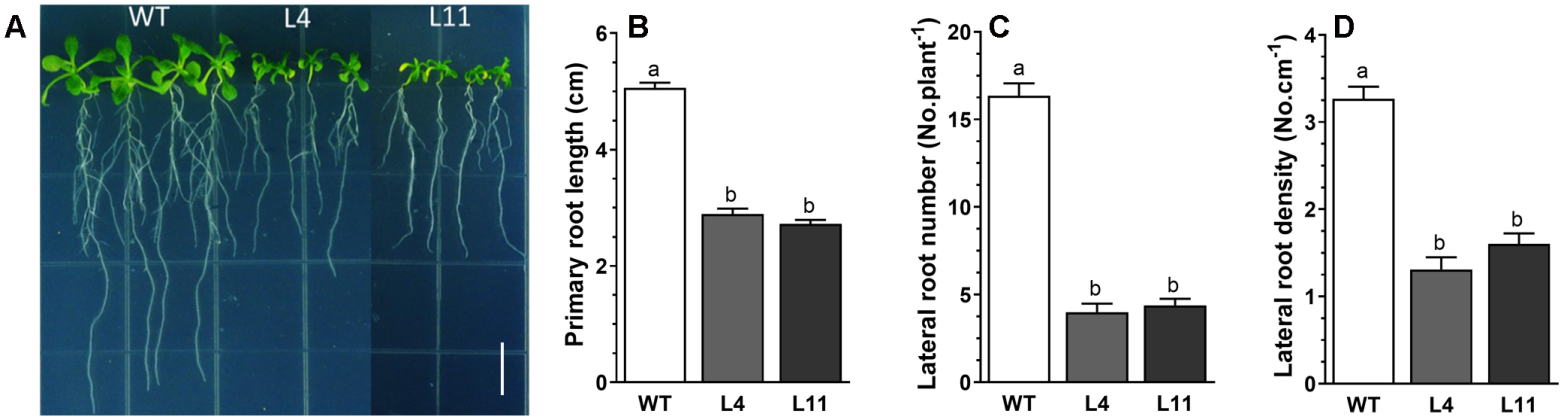
Root phenotypes of *MtHB2*-expressing transgenic *Arabidopsis* plants. Six-day-old seedlings were transferred to vertical plates and were grown for 4 days before photographed **(A)** and analysis for root growth and development including primary root length **(B)**, lateral root number **(C)** and density **(D)**. Data represent the mean ± SE; n≥18. Different letters shown in the error bars indicate significant differences among WT and transgenic lines (L4 and L11) at P < 0.05. Bar=1 cm.

Upon expressing MtHB2 in transgenic Arabidopsis under the control of the cauliflower mosaic virus (CaMV) 35S promoter, several independent transgenic lines were obtained, as confirmed by PCR analysis. The expression levels of MtHB2 in the selected lines, determined through RT-qPCR, are illustrated in **Supplementary Figure S1**. Notably, the transgenic lines exhibited shorter primary roots compared to wild-type plants under normal growth conditions with full nutrient supplementation (**Figure 1A**). Furthermore, the expression of *MtHB2* resulted in decreased lateral root number and density in the transgenic plants compared to wild-type plants (**Figure 1B**). The extent of these phenotypic changes appeared to be positively correlated with the level of *MtHB2* expression. Considering the crucial role of auxin in root growth and development, the observed alterations in the transgenic lines suggest a potential disruption in auxin transport and distribution. Further analysis revealed an increased MtHB2 transcript in response to IAA treatment, peaking at 2 hours and declining thereafter (**Supplementary Figure S2**). The presence of auxin-responsive elements in the promoter sequence of *MtHB2* suggests its involvement in auxin response mechanisms.

### 3.2 Expression of *MtHB2* alters root response to auxin

**Figure 2:**
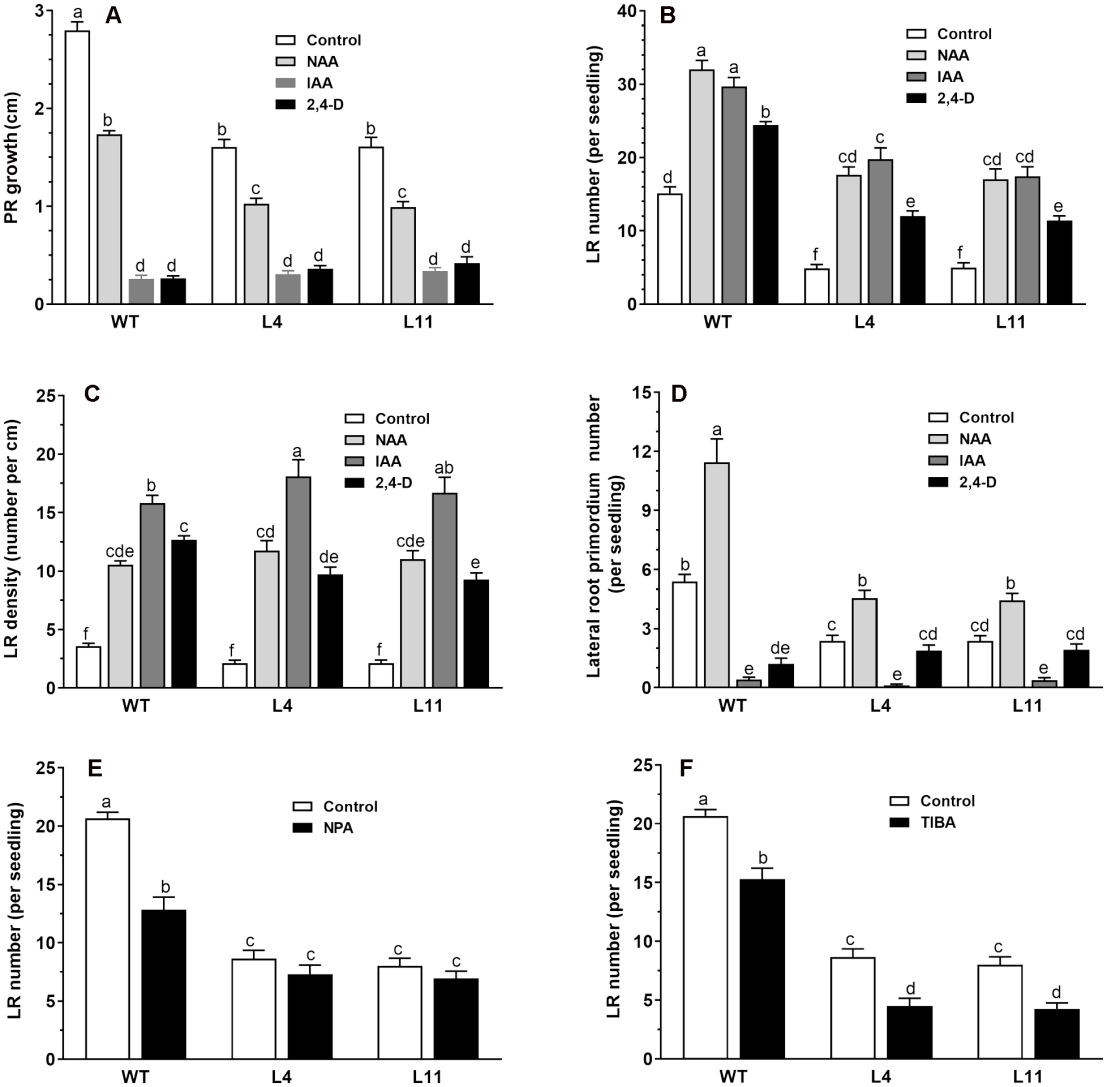
Comparison of root response between the wild type and *MtHB2*-expressing transgenic plants to auxins and auxin transport inhibitors. Effects of 0.1 µM NAA, 1 µM IAA, and 0.05 µM 2,4-D on root system architecture of the wild type and *MtHB2*-expressing transgenic plants are shown in **(A-D)**. Additionally, effects of 0.5 µM NPA and 0.1 µM TIBA on lateral root formation of wild type and MtHB2-expressing transgenic plants are depicted in **(E, F)**. Data represent the mean ± SE; n>15. Different letters within the error bars indicate significant differences at P < 0.05.

To investigate the altered auxin responses in the transgenic lines, we compared the root responses of wild-type and transgenic seedlings expressing *MtHB2* to various exogenous auxin analogs with distinct transport mechanisms. Six-day-old wild-type and transgenic seedlings were subjected to different auxin analog treatments, and root phenotypes were evaluated after a 4-day incubation period. In the absence of auxin analogs, the primary root length in transgenic lines was notably shorter than in wild-type plants, and the growth of primary roots in both genotypes was inhibited by the auxin analogs. While NAA induced similar reductions in primary root growth in both wild-type and transgenic seedlings, IAA and 2,4-D had a greater inhibitory effect on primary root growth in wild-type plants compared to the transgenic lines. For instance, treatment with IAA and 2,4-D inhibited PR growth of WT by 91%, while the same treatment reduced PR growth of transgenic line L11 by 79% and 74%, respectively (**Figure 2A**). Moreover, the transgenic lines exhibited a lower lateral root number than wild-type plants in the absence of auxin analogs, yet treatment with these analogs led to a more increase rate of lateral root number in transgenic lines than that of wild-type plants (**Figure 2B**). Treatment with IAA increased the number of lateral roots per seedlings of WT plants by 93%, while this percentage became 300% and 256% for transgenic line L4 and L11, respectively. This differential response indicates that the expression of *MtHB2* renders the transgenic plants more sensitive to auxin. Similar trends were observed in the effects of IAA treatment on lateral root density (**Figure 2C**). IAA increased the LR density of WT plants by 357%, while this percentage was about 800% for transgenic line L4. Additionally, the investigation on lateral root primordia development revealed significant differences between wild-type and transgenic seedlings in response to IAA and 2,4-D treatments, further indicating the altered auxin response in transgenic plants (**Figure 2D**).

### 3.3 Expressing *MtHB2* altered sensitivity to auxin transport inhibitors

The contrasting responses of lateral roots to exogenously applied auxin analogs between wild-type (WT) and transgenic plants imply a potential association between MtHB2 and auxin translocation and distribution in roots. To delve deeper into whether the reduced lateral root phenotype of transgenic seedlings is linked to impaired polar auxin transport, the impact of auxin efflux inhibitors NPA and TIBA on lateral root number and density in both WT and transgenic plants was assessed.

Treatment with NPA and TIBA resulted in a significant reduction in lateral root number in both WT and transgenic plants (**Figures 2E, F**). The inhibitory effects were more pronounced in WT plants than in transgenic plants. For instance, treatment with 0.5 μM NPA decreased lateral root number in WT, L4, and L11 by 38%, 15%, and 16%, respectively (**Figure 2E**). In contrast, a greater inhibitory effect was observed in transgenic seedlings compared to WT upon application of TIBA (**Figure 2F**).

### 3.4 Impact of *MtHB2* on auxin accumulation and efflux protein expression in roots of transgenic seedlings

**Figure 3:**
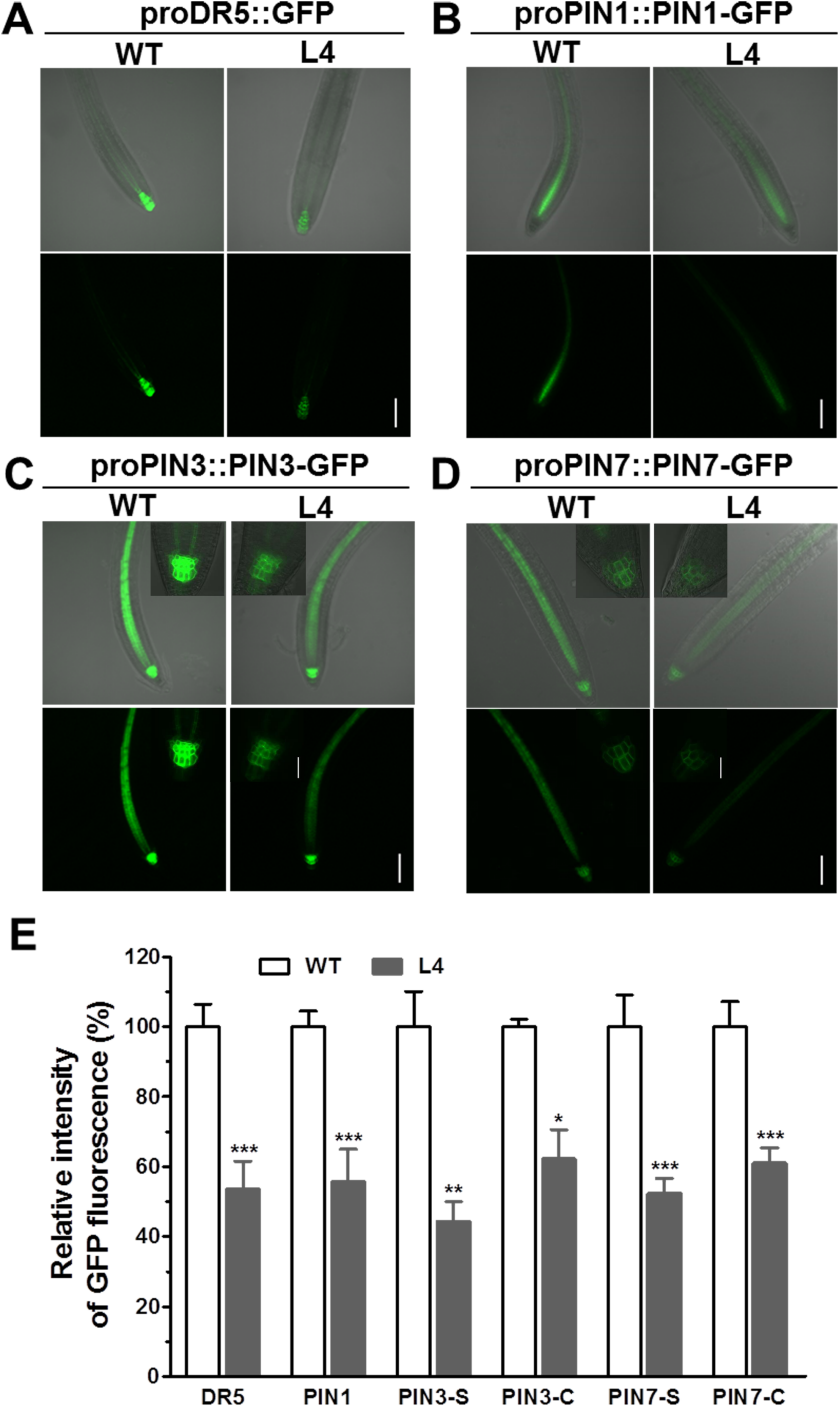
Comparison of pro*DR5::GFP* and auxin efflux protein expression in root apical tissues of 6-day-old seedlings of wild type and *MtHB2*-expressing transgenic *Arabidopsis* plants. GFP images including pro*DR5::GFP* **(A)**, pro*PIN1::PIN1-GFP* **(B)**, pro*PIN3::PIN3-GFP* **(C)** and pro*PIN7::PIN7-GFP* **(D)** were captured with a confocal microscope at the same settings to enable comparison of image strength. Bars=100 µm. Representative images of PIN3-C and PIN7-C were added in corresponding positions and bars=20 µm. **(E)** Quantification of GFP fluorescence by image analysis of confocal sections. The relative intensity in wild type (WT) is considered as 100%. PIN3-S and PIN7-S represent PIN3 and PIN7 expression in stele, and PIN3-C and PIN7-C represent PIN3 and PIN7 expression in columella cells. Data represent the mean ± SE; n≥10. Asterisks indicate statistically significant difference between wild-type and transgenic lines lines according to T test. *P <0.05, **P <0.01, ***P <0.001.

**Figure 4:**
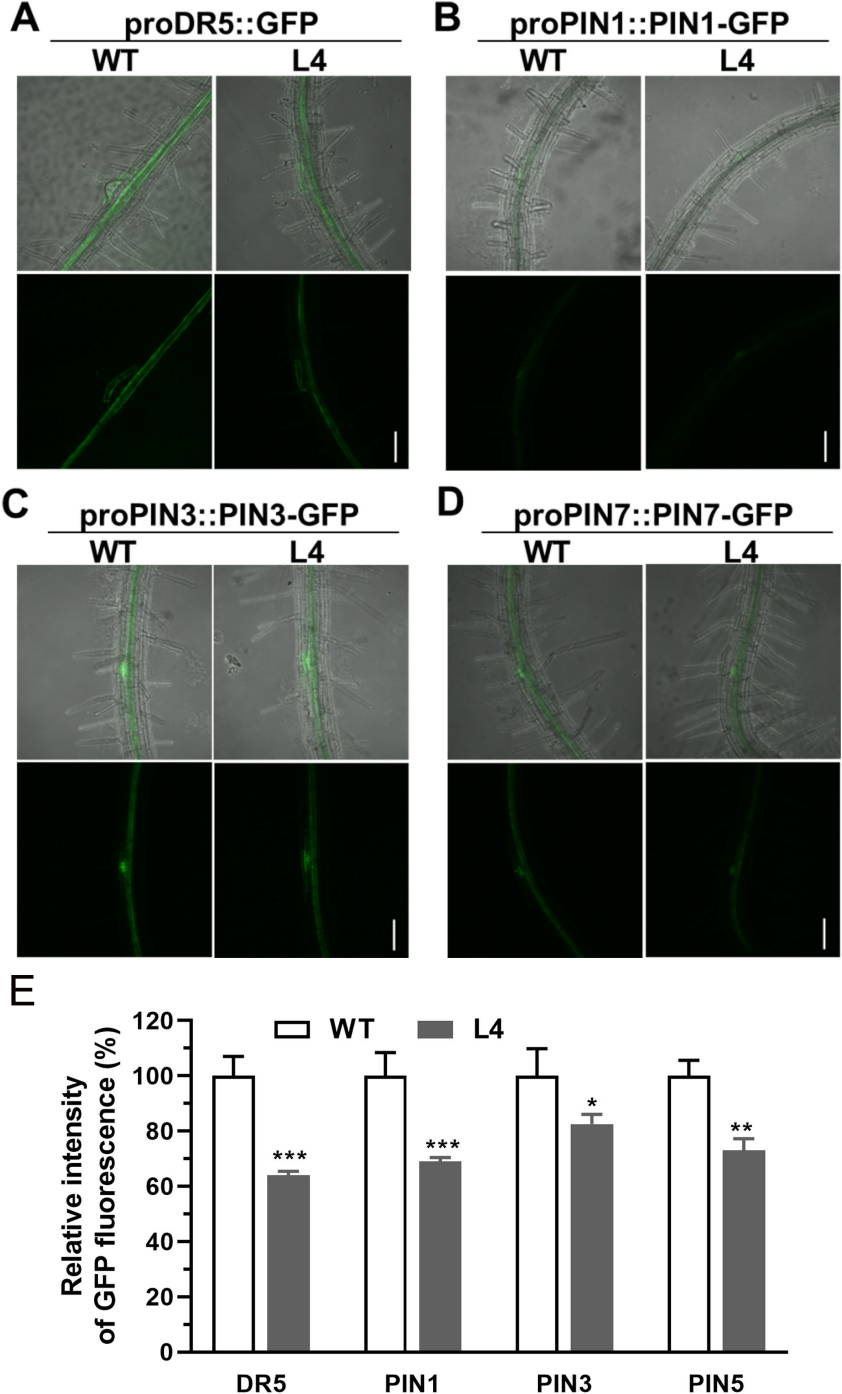
Comparison of pro*DR5::GFP* and auxin efflux protein expression in root vascular tissues of 6-day-old seedlings of wild type and *MtHB2*-expressing transgenic *Arabidopsis* plants. GFP images including proDR5::GFP **(A)**, pro*PIN1::PIN1-GFP* **(B)**, pro*PIN3::PIN3-GFP* **(C)** and pro*PIN7::PIN7-GFP* **(D)** were captured with a confocal microscope at the same settings to enable comparison of image strength. Bars=100 µm. **(E)** Quantification of GFP fluorescence by image analysis of confocal sections. The relative intensity in wild type (WT) is considered as 100%. Data represent the mean ± SE; n≥10. Asterisks indicate statistically significant difference between wild-type and transgenic lines lines according to T test. *P <0.05, **P <0.01, ***P <0.001.

To ascertain the effects of *MtHB2* expression on auxin response, accumulation, and polar auxin transport in transgenic seedlings, we introduced the auxin response reporter *pro*DR5::GFP and auxin efflux carriers *pro*PIN1::PIN1-GFP, *pro*PIN3::PIN3-GFP, and *pro*PIN7::PIN7-GFP into transgenic seedling L4 through crossing. Confocal microscopy examination of GFP fluorescence in 6-day-old seedlings revealed significant reductions in DR5 activity in root tips, including quiescent center (QC) cells, columella cells, and certain vascular cells in transgenic seedlings. This suggests that the expression of *MtHB2* leads to diminished auxin accumulation or response in the root tips of transgenic lines. Similarly, the expression of PIN1 in the stele and PIN3 and PIN7 in the stele and columella was notably decreased in transgenic seedling root apices, potentially contributing to the attenuated DR5 activity (**Figure 3**). Additionally, weaker GFP fluorescence was observed in vascular tissues of transgenic seedling roots (**Figure 4**).

**Figure 5:**
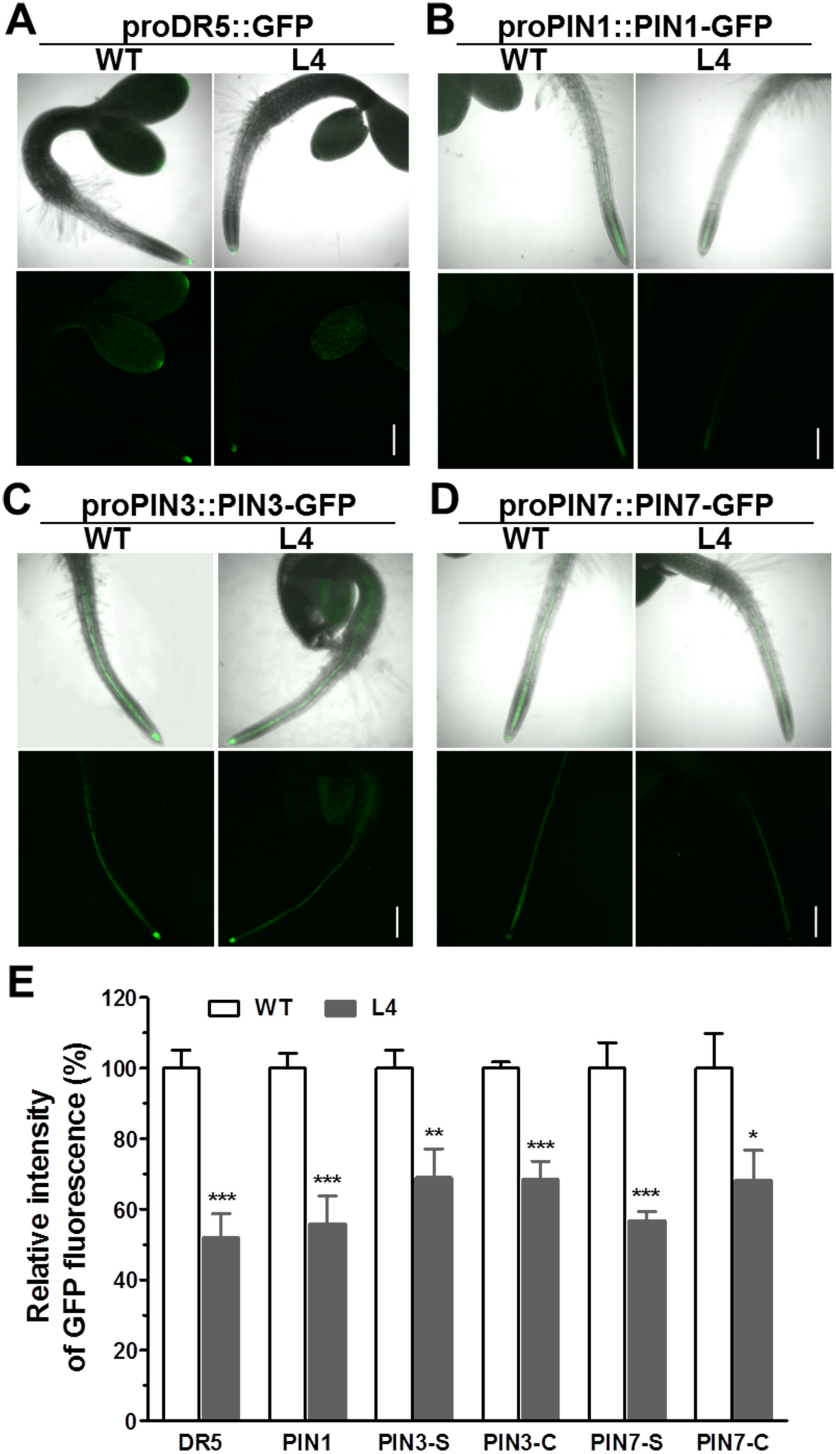
Comparison of pro*DR5::GFP* and auxin efflux protein expression in roots of 2-day-old seedlings of wild type and *MtHB2*-expressing transgenic *Arabidopsis* plants. GFP images including proDR5::GFP **(A)**, pro*PIN1::PIN1-GFP* **(B)** and pro*PIN3::PIN3-GFP* **(C)** and pro*PIN7::PIN7-GFP* **(D)** were captured with a confocal microscope at the same settings to enable comparison of image strength. Bars=200µm. **(E)** Quantification of GFP fluorescence by image analysis of confocal sections. The relative intensity in wild type (WT) is considered as 100%. PIN3-S and PIN7-S represent PIN3 and PIN7 expression in stele, and PIN3-C and PIN7-C represent PIN3 and PIN7 expression in columella cells. Data are mean ± SE, n≥10. Asterisks indicate statistically significant differences between wild-type and transgenic lines according to T test. *P <0.05, **P <0.01, ***P <0.001.

For a more in-depth investigation into whether *MtHB2* expression impairs auxin response, accumulation, and polar auxin transport in transgenic seedlings at earlier developmental stages before lateral root initiation, GFP fluorescence of 2-day-old seedlings was examined. Consistently, the expression levels of pro*DR5::GFP*, pro*PIN1::PIN1-GFP*, pro*PIN3::PIN3-GFP*, and pro*PIN7::PIN7-GFP* were all significantly diminished in the root tips of transgenic seedlings (**Figure 5**). These findings suggest that reduced auxin accumulation in roots due to impaired polar auxin transport may contribute to the decreased formation of lateral roots and slower primary root growth in transgenic seedlings.

### 3.5 Comparison of gene expression profiles in wild-type and *MtHB2*-expressing transgenic plants

**Figure 6:**
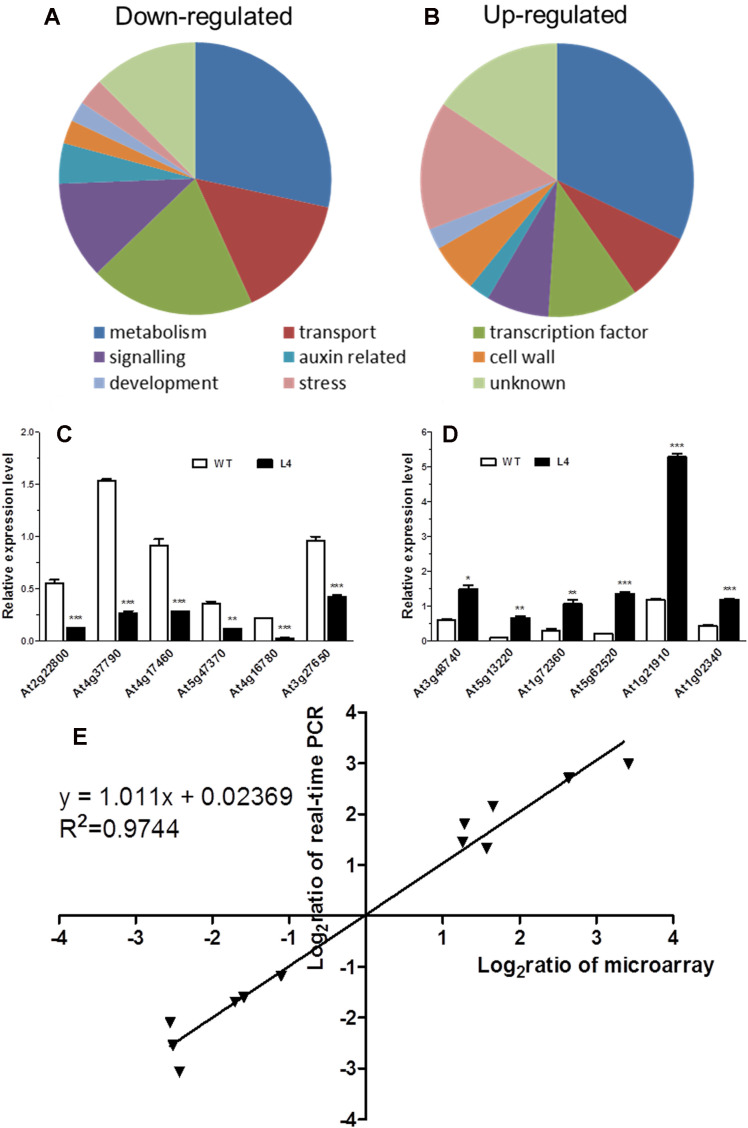
Global analysis of gene expression in *MtHB2*-expressing transgenic *Arabidopsis*. Predicted functions of the proteins encoded by down-regulated genes **(A)** and up-regulated genes **(B)**. Relative expression in the wild type and the transgenic line of six down-regulated genes **(C)** and six up-regulated genes **(D)** selected from the microarray data was confirmed by RT-qPCR. **(E)** Correlation between data obtained from microarray and RT-qPCR data. Data represent the mean ± SE of three replicates. *P <0.05, **P <0.01, ***P <0.001.

To delve deeper into the mechanisms underlying the alteration of root phenotype by *MtHB2* expression, a comparative transcriptomic analysis was conducted between *Arabidopsis* transgenic (*35S:MtHB2*) and wild-type plants. Root tissues from 18-day-old seedlings of the transgenic line and wild-type plants grown vertically on agar plates under standard conditions were used for total RNA extraction. The data was then normalized to evaluate relative changes in gene expression in the transgenic line (L4) compared to wild-type plants. Out of 22,746 genes analyzed, 250 genes were down-regulated while 243 genes were up-regulated by more than 2-fold (**Supplementary Table S1**, **Figures 6A, B**).

The validity of the microarray data was confirmed via RT-qPCR analysis. Twelve genes with diverse expression profiles were selected for analysis, all of which exhibited expression patterns consistent with the microarray results (**Figures 6C, D**). A high level of agreement was observed between the findings obtained from both methods (**Figure 6E**).

Subsequently, a detailed examination of the differentially expressed genes, particularly those related to auxin, was carried out (**Table 1**). In line with the reduced DR5 expression, several auxin-responsive genes, including *Aux/IAA* genes, *SAUR* genes, and *GH3* genes, displayed decreased expression levels. Notably, genes such as *IAA3*, *IAA14*, *SAUR31*, *SAUR59*, *SAUR72*, *SAUR76*, and *GH3.17* exhibited marked reductions in expression (**Table 1**). Furthermore, alterations were observed in genes involved in auxin homeostasis. The down-regulation of auxin biosynthesis genes (*TAA1*, *YUC6*, *CYP79B2/B3*) and up-regulation of IAA-amino acid hydrolase *ILL6* and MeIAA methyl esterases AtMES1, 16 (**Table 1**) hinted at potential impairment of auxin accumulation in the roots of transgenic lines. Additionally, three auxin-responsive genes from the *LATERAL ORGAN BOUNDARIES* (*LBD*) family (*LBD4*, *LBD16*, and *LBD25*) showed reduced expression in the transgenic lines (**Table 1**). Conversely, genes that are typically up-regulated by auxin, such as *PLDζ2*, *PBP*, *ACS6*, and *STZ*, were found to be down-regulated (**Table 1**). Surprisingly, the expression of auxin transport genes in the transgenic lines remained unaffected (**Table 1**), contrasting with the observed down-regulation of *PIN1*, *PIN3*, and *PIN7* in the root apices of the transgenic lines as visualized by GFP fluorescence.

**Table 1:** Auxin-related genes which change their transcript levels in roots of transgenic *Arabidopsis* plants expressing *MtHB2*.

Gene ID^a^	Gene symbol^b^	Description^c^	Ratio^d^
Auxin transport genes			
At2g38120	*AUX1*	auxin influx carrier	0.6950
At1g77690	*LAX3*	auxin influx carrier	0.5949
At1g73590	*PIN1*	auxin efflux carrier	0.8183
At5g57090	*PIN2*	auxin efflux carrier	0.9740
At1g70940	*PIN3*	auxin efflux carrier	0.8905
At2g01420	*PIN4*	auxin efflux carrier	0.7997
At1g23080	*PIN7*	auxin efflux carrier	1.4572
At2g36910	*ABCB1*	auxin efflux carrier	0.8016
At2g47000	*ABCB4*	auxin efflux carrier	0.8826
At3g28860	*ABCB19*	auxin efflux carrier	0.8045
At2g34650	*PINOID*	protein serine/threonine kinase	0.7857
At1g53700	*WAG1*	protein serine/threonine kinase	0.6299
At3g14370	*WAG2*	protein serine/threonine kinase	1.1598
At1g25490	*RCN1*	protein phosphatase type 2A regulator	0.8046
Auxin biosynthesis genes			
At1g70560	*TAA1*	tryptophan aminotransferase	0.5590
At5g25620	*YUC6*	YUCCA	0.4649
At4g39950	*CYP79B2*	cytochrome p450 enzyme	0.5780
At2g22330	*CYP79B3*	cytochrome p450 enzyme	0.5987
At4g31500	*CYP83B1*	cytochrome p450 enzyme	0.6560
At2g23620	*MES1*	methyl esterase 1	3.7146
At4g16690	*MES16*	methyl esterase 16	3.4728
At1g44350	*ILL6*	IAA-amino acid conjugate hydrolase	2.1618
Auxin responsive genes			
At4g14550	*IAA14*	AUX/IAA family	0.3794
At1g04240	*IAA3*	AUX/IAA family	0.4121
At5g53590	*SAUR30*	SAUR like	0.4154
At4g00880	*SAUR31*	SAUR like	0.3881
At4g31320	*SAUR37*	SAUR like	0.3990
At3g60690	*SAUR59*	SAUR like	0.3483
At3g12830	*SAUR72*	SAUR like	0.3476
At5g20820	*SAUR76*	SAUR like	0.4283
At1g72430	*SAUR78*	SAUR like	0.3232
At1g28130	*GH3.17*	GH3 like	0.5031
At3g05630	*PLDζ2*	phospholipase D	0.4343
At5g54490	*PBP1*	PINOID-BINDING PROTEIN 1	0.4540
At3g27650	*LBD25*	LOB DOMAIN-CONTAINING PROTEIN	0.1857
At1g31320	*LBD4*	LOB DOMAIN-CONTAINING PROTEIN	0.4846
At2g42430	*LBD16*	LOB DOMAIN-CONTAINING PROTEIN	0.5142
At1g27730	*STZ*	salt tolerance zinc finger	0.3500
At4g11280	*ACS6*	ACC synthase related	0.3608

a TAIR locus number.

b Gene name.

c Gene annotation in the TAIR database.

d The ratio between transcript levels in transgenic (35S:MtHB2) plants relative to those in WT plants. Data are mean of two biological replicates.

Moreover, several genes known to act as repressors of jasmonate (JA) response were found to be up-regulated in the transgenic lines. The elevated transcript levels of *CYP94B3*, responsible for encoding a JA-Ile-12-Hydroxylase, and five *JAZ* (*JASMONATE-ZIM DOMAIN*) family genes suggested potential attenuation of JA signaling in the transgenic lines (Kazan and Manners, 2012). Additionally, there were indications of diminished cytokinin signaling, as evidenced by the down-regulation of three cytokinin signaling genes, including *CRE1* (*CYTOKININ RESPONSE 1*), *ARR7* (*RESPONSE REGULATOR 7*), and *ARR11* (Fukaki and Tasaka, 2009) (**Table 2**). The transcript levels of *Arabidopsis* HD-Zip II members homologous to *MtHB2* were notably reduced, particularly the HD-ZIP II β genes (*HAT9* and *HAT22*) and γ genes (*HAT1*, *HAT2*, and *ATHB2*) within the transgenic plants (**Table 3**).

**Table 2:** Jasmonate acid and cytokinin signalling related genes which change their transcript levels in roots of transgenic *Arabidopsis* plants expressing *MtHB2*.

Gene ID^a^	Gene symbol^b^	Description^c^	Ratio^d^
At1g74950	*JAZ2*	Jasmonate-Zim-domain transcription repressor	2.3071
At1g72450	*JAZ6*	Jasmonate-Zim-domain transcription repressor	2.4441
At2g34600	*JAZ7*	Jasmonate-Zim-domain transcription repressor	4.0930
At1g30135	*JAZ8*	Jasmonate-Zim-domain transcription repressor	8.5900
At5g13220	*JAZ10*	Jasmonate-Zim-domain transcription repressor	10.7057
At3g48520	*CYP94B3*	cytochrome p450 enzyme	5.5497
At2g01830	*CRE1*	CYTOKININ RESPONSE 1	0.4554
At1g19050	*ARR7*	RESPONSE REGULATOR 7	0.3250
At1g67710	*ARR11*	RESPONSE REGULATOR 11	0.4488

a TAIR locus number.

b Gene name.

c Gene annotation in the TAIR database.

d The ratio between transcript levels in transgenic (35S:MtHB2) plants relative to those in WT plants. Data are mean of two biological replicates. Data are mean of two biological replicates.

**Table 3:** *Arabidopsis* HD-Zip II members which change their transcript levels in roots of transgenic *Arabidopsis* plants expressing *MtHB2*.

Gene ID^a^	Gene symbol^b^	Description^c^	Ratio^d^
At2g22800	*HAT9*	HD-ZIP II transcription factor	0.1708
At4g37790	*HAT22*	HD-ZIP II transcription factor	0.1752
At4g17460	*HAT1*	HD-ZIP II transcription factor	0.2964
At5g47370	*HAT2*	HD-ZIP II transcription factor	0.3062
At4g16780	*ATHB2*	HD-ZIP II transcription factor	0.3323
At3g60390	*HAT3*	HD-ZIP II transcription factor	0.8132
At2g44910	*ATHB4*	HD-ZIP II transcription factor	0.8506
At2g01430	*ATHB17*	HD-ZIP II transcription factor	0.4434
At1g70930	*ATHB18*	HD-ZIP II transcription factor	0.6441
At5g06710	*HAT14*	HD-ZIP II transcription factor	0.4965

a TAIR locus number.

b Gene name.

c Gene annotation in the TAIR database.

d The ratio between transcript levels in transgenic (35S:MtHB2) plants relative to those in WT plants. Data are mean of two biological replicates.

Data are mean of two biological replicates.

## 4 Discussion

Evidence is emerging that the HD-Zip I and II transcription factors play a regulatory role in the response of plants to environmental and hormonal cues (Harris et al., 2011; Gong et al., 2019). Several studies have shown that HD-Zip II proteins are involved in stress responses, leaf chlorophyll, flowering induction, shade avoidance responses, and the regulation of various plant developmental processes (Oh et al., 2013; Carabelli et al., 2013; Preciado et al., 2022). Previous reports have indicated that HD-Zip II proteins modulate root growth and development, as shown by phenotypic observations in overexpression transgenic plants (Ruberti et al., 2012; He et al., 2020). However, mechanisms through which HD-Zip II proteins regulate root growth and development warrant further investigation.

In the current study, we discovered that the expression of a gene, *MtHB2*, encoding an HD-ZIP II protein in *Medicago truncatula*, when introduced into *Arabidopsis*, led to changes in root growth and development in the transgenic seedlings. It was further demonstrated that the expression of *MtHB2* altered root auxin response and transport, as supported by microarray analysis of wild-type and transgenic plants. It has been identified that *MtHB2* is closely related to *Arabidopsis* HD-Zip II β genes (*HAT22* and *HAT9*) (Song et al., 2012). *HAT22* is up-regulated by cytokinin, and overexpression of *HAT22* reduces chlorophyll contents and accelerates leaf senescence (Köllmer et al., 2011). It’s interesting that the phenotype of over-expression *MtHB2* was not consistent with the above studies. In our investigation, we observed that the expression of *MtHB2* was promptly up-regulated by IAA (**Supplementary Figure S2**), and transgenic *Arabidopsis* seedlings expressing *MtHB2* displayed root phenotypes characterized by shorter primary root length, fewer lateral roots, and lower root density compared to wild-type seedlings (**Figure 1**). Studies have reported that the expression of HD-Zip II genes is mutually regulated, meaning heightened expression of one gene leads to a down-regulation of the others (Sorin et al., 2009). Our microarray data, showing reduced transcript levels of *Arabidopsis* HD-Zip II members homologous to *MtHB2* in the transgenic lines (**Table 3**), align with previously reported findings.

Previous studies have shown that IAA can rescue the lateral root phenotype of *ATHB2*-overexpressing seedlings (Steindler et al., 1999), and that primary root elongation in 35S:HAT2 seedlings is less inhibited by NAA than in wild-type plants (Sawa et al., 2002). We observed that treatment with three types of auxin (IAA, NAA, and 2,4-D) exhibited a more pronounced stimulatory effect on lateral root development and a lesser inhibitory effect on primary root growth in the transgenic plants than in wild-type plants, partially rescuing the root phenotype of transgenic plants. These findings suggest that auxin accumulation in the root may be reduced in *MtHB2*-expressing *Arabidopsis* seedlings.

The study revealed a more pronounced stimulatory effect of IAA and NAA on lateral roots of transgenic seedlings compared to 2,4-D, suggesting potential involvement of auxin efflux facilitators in the MtHB2-dependent root phenotype. Additionally, the reduced sensitivity of transgenic seedlings to the auxin transport inhibitor NPA indicates impaired auxin transport, leading to diminished auxin accumulation in the root. Conversely, the heightened sensitivity to TIBA could be attributed to differences in the modes of action between NPA and TIBA, where TIBA, being a competitive auxin efflux inhibitor, exerts a stronger effect than the non-competitive NPA, and may also possess auxin antagonistic properties (Paponov et al., 2008).

In this investigation, transgenic lines expressing *MtHB2* exhibited dampened expression of *PIN1*, *PIN3*, and *PIN7*, key players in auxin polar transport, along with reduced DR5 activity in their root apices (**Figures 3**–**5**). These outcomes suggest that the over-expression of *MtHB2* affected auxin homeostasis, probably by inhibiting its synthesis and accumulation.

Previous research has indicated that increased *ATHB4* activity leads to a reduction in the expression of three auxin-responsive genes - *SAUR15*, *SAUR68*, and *IAA1* (Sorin et al., 2009). Consistent with this, the microarray analysis in this study revealed a down-regulation of several auxin-responsive genes in transgenic seedlings expressing *MtHB2* (**Table 1**), aligning with the hypothesis of decreased auxin accumulation in the roots of these transgenic plants. Notably, among the down-regulated auxin-responsive genes due to *MtHB2* overexpression were *LBD4*, *LBD16*, and *LBD25*, which are known to play crucial roles in auxin signaling and lateral root formation (Mangeon et al., 2011; Zhang et al., 2020).

Further analysis of the promoter sequence of *LBD25* revealed a putative target site for *MtHB2* binding, suggesting a potential regulatory mechanism for this gene. Moreover, the down-regulation of auxin biosynthesis genes like *TAA1*, *YUC6*, *CYP79B2/B3* indicates impaired auxin accumulation in the roots of transgenic lines, while the up-regulation of IAA-amino acid hydrolase *ILL6* and MeIAA methyl esterases *AtMES1* and *AtMES16* may contribute to active auxin levels and aid in auxin homeostasis. Additionally, the discrepancy between the visualized activity of PIN1, PIN3, and PIN7 proteins and their gene expressions in transgenic seedlings hints at potential posttranslational regulation mechanisms affecting their levels.

The crosstalk between auxin and other plant hormones, such as cytokinin and jasmonic acid, is crucial for orchestrating root development. For instance, cytokinin can negatively impact lateral root formation by interfering with auxin transport or response, while jasmonic acid influences primary root growth and lateral root formation through both auxin-independent and dependent mechanisms. The study suggests that the attenuation of jasmonic acid and cytokinin signaling in transgenic lines, as indicated by the gene expression changes, may be a plant response to counterbalance the effects of *MtHB2*-mediated alterations in auxin accumulation on root growth and development.

In summary, the study demonstrates that the over-expression of *MtHB2* results in significant alterations in root phenotype, characterized by shorter primary roots and reduced lateral root number and density compared to wild-type plants. These changes are associated with impaired auxin transport and accumulation in the root tips, highlighting the role of MtHB2, an HD-Zip II protein, in regulating auxin-dependent root growth and development.

## Data Availability

The raw data supporting the conclusions of this article will be made available by the authors, without undue reservation.
