# Supplementary material for: A Medicago truncatula HD-ZIP gene MtHB2 is involved in modulation of root development by regulating auxin response
Source: Front Plant Sci. 2024 Sep 19;15:1466431. doi: 10.3389/fpls.2024.1466431 (PMC11446790; doi:10.3389/fpls.2024.1466431)
Supplement: Supplementary file 1 [file DataSheet1.docx]

**Supplementary data**

**Fig. S1** RT-qPCR analysis of *MtHB2* expression level in wild-type (WT) and transgenic *Arabidopsis* plants. Data represent the mean±SE of three biological replicates.

**Fig. S2** RT-qPCR analysis of *MtHB2* expression in response to auxin (10µM IAA) in *Medicago* *truncatula*. Data represent the mean±SE of three biological replicates.

**Table S1.** The primers designed for RT-qPCR

| Gene | Forward sequence primer (5'-3') | Reverse sequence primer (5'-3') |
| --- | --- | --- |
| *MtHB2* | GTGGATTGCGAGTTCTTG | TTGGAGCCATAGTGAAGG |
| *MtActin* | ACGAGCGTTTCAGATG | ACCTCCGATCCAGACA |
| *AtActin11* | TGTTCTTTCCCTCTACGCT | CCTTACGATTTCACGCTCT |
| At2g22800 | GACAATCCTCCGTTTACAAGC | GACTCTTCGCCACCGTCTC |
| At4g37790 | CACTGTGGACCATCGTTTCAT | CGGCACCAGTCTTGATCTTG |
| At4g17460 | CGGAGACTTCAGAAAGAGGC | GACCAGCACAAGCAACCC |
| At5g47370 | CGAAGCAGAAGCAAGCATT | AAGAACTCGCAGTCTACCTCC |
| At4g16780 | TATTCGCACCCTATTTTCCG | CGCTCCAACGCAGCCATA |
| At3g27650 | ACCTACCCGACGAAGCAACA | AAACGCAGCCGACTCAACC |
| At3g48740 | CTCTAAGCATAATCAGGACGGT | AGCACATTCGGGAAAGCA |
| At5g13220 | CAAACCAACAACGCTCCTA | CGAACGAGATTTAGCCGAT |
| At1g72360 | AAGATGGGCGGCTGAGATA | CTTTCCAGAGGATTCGTTAGG |
| At5g62520 | GGGCTTATGGAGTTGCTTG | CACCAACCGTATTTCACCTTA |
| At1g21910 | TTCATCAGCCGTCTCGTCA | AAACTCCAAAGCGGAATGTC |
